# Dumbbell-shaped thoracic epidural capillary hemangioma mimicking schwannoma: a case report

**DOI:** 10.1186/s13019-026-04461-7

**Published:** 2026-06-21

**Authors:** Srikanth Thiyagarajan, Sri Rahul, Mohamed Naleer, Jeyaselva Senthilkumar, Manisha Rathore

**Affiliations:** 1 Department of general surgery, SRM Medical College Hospital and Research Centre, Faculty of medicine and health sciences, SRM institute of science and Technology, kattankulathur, Chengalpattu, Tamil Nadu India; 2 Department of Neurosurgery, SRM Medical College Hospital and Research Centre, Faculty of medicine and health sciences, SRM institute of science and Technology, kattankulathur, Chengalpattu, Tamil Nadu India

**Keywords:** Spinal tumor, Dumbbell tumor, Capillary hemangioma, Epidural hemangioma, Thoracotomy

## Abstract

**Background:**

Spinal epidural hemangiomas are rare benign vascular lesions. A dumbbell configuration defined by extension through the neural foramen with both intraspinal and extraspinal components is most commonly associated with schwannomas. Dumbbell-shaped capillary hemangiomas are exceptionally rare and can pose significant diagnostic challenges.

**Case presentation:**

A 66-year-old woman presented with a one-year history of back pain and progressive bilateral lower-limb weakness that progressed to a bedridden state. Imaging revealed a dumbbell-shaped lesion extending from D3 to D5 with a large paravertebral component measuring 4.3 × 3.6 × 5.7 cm. A combined D3–D5 laminectomy and right posterolateral thoracotomy was performed. The tumor was markedly hypervascular intraoperatively, and gross total resection was achieved. Recent coronary artery bypass grafting and dual antiplatelet therapy increased the operative hemorrhagic risk. Postoperatively, the patient demonstrated neurological improvement with restoration of bladder control and regained ambulation with a walker.

**Conclusions:**

Dumbbell capillary hemangiomas can mimic schwannomas radiologically yet carry substantial hemorrhagic risk. Multidisciplinary surgical planning is essential for safe management.

## Introduction

Vascular malformations constitute approximately 2–7% of spinal space-occupying lesions and include cavernous angiomas, arteriovenous malformations, capillary telangiectasias, and capillary hemangiomas [1, 2]. Capillary hemangiomas most commonly involve cutaneous or mucosal tissues of the head and neck in children, whereas spinal extraosseous involvement is distinctly uncommon [3].

Primary spinal epidural hemangiomas account for approximately 4% of epidural spinal lesions [4, 5]. Histologically, they are classified into cavernous and capillary types. Spinal epidural capillary hemangiomas are exceedingly rare, with fewer than 20 cases reported, and only a small fraction demonstrate a dumbbell configuration [6, 7].

The term dumbbell tumor refers to lesions extending through the intervertebral foramen, connecting intraspinal and extraspinal compartments. This morphology is classically associated with schwannomas. Non-neurogenic dumbbell tumors are rare, and capillary hemangioma presenting in this form represents an exceptional diagnostic entity [8, 9]. We report a thoracic dumbbell capillary hemangioma in a high-risk cardiac patient, emphasizing diagnostic challenges, surgical decision-making, and outcome.

## Case presentation

A 60-year-old female presented with a two-month history of progressive ascending weakness of both lower limbs, which progressed to complete paraplegia over six weeks. She also reported sensory loss below the D6 dermatome with urinary retention and constipation, suggestive of thoracic spinal cord compression. Neurological examination revealed spastic paraplegia with increased tone and absent voluntary motor activity in both lower limbs, with hypoesthesia below the D6 level and neurogenic bladder and bowel dysfunction.

**Fig. 1:**
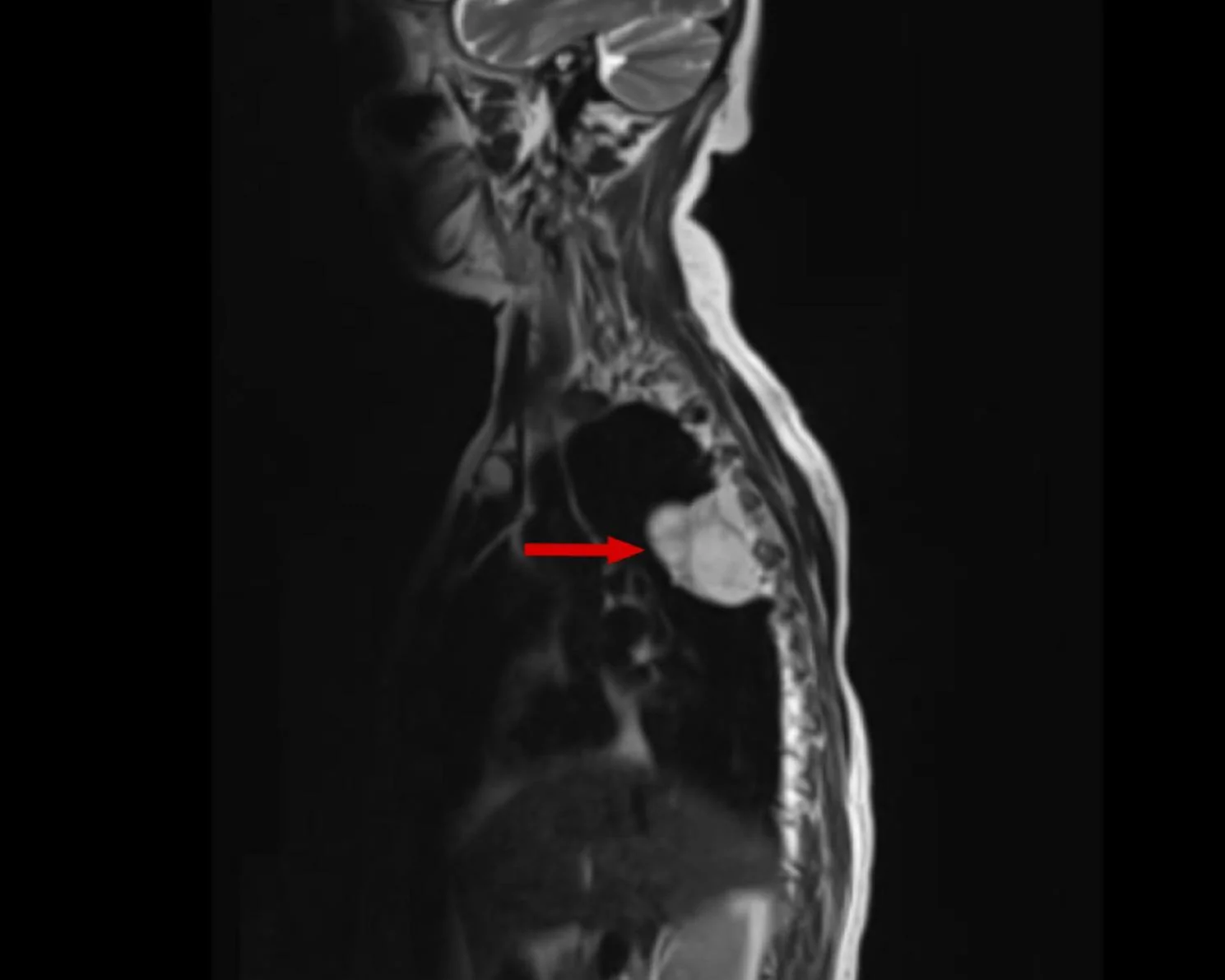
Preoperative imaging of the Throracic Dumbell Capillary Hemangioma. Sagittal T2-weighted MRI showing the hyperintense, lobulated, dumbbell-shaped epidural mass (arrow) causing severe compression of the spinal cord at the D3-D5 level

**Fig. 2:**
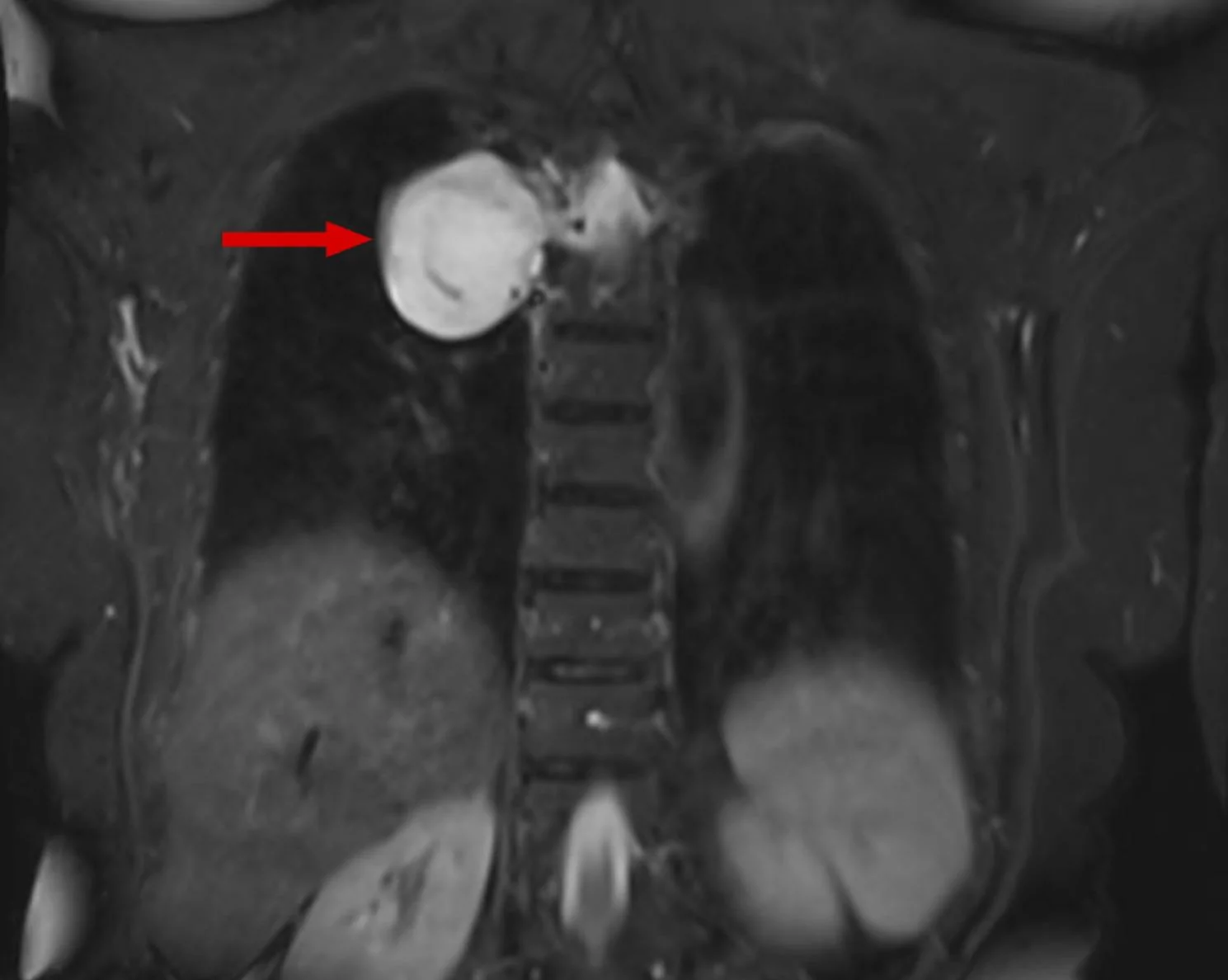
Post-contrast MRI demonstrating the intensely enhancing, dumbbell-shaped lesion (Arrow), highlighting the large extraspinal paravertebral component and the smaller intraspinal component

**Fig. 3:**
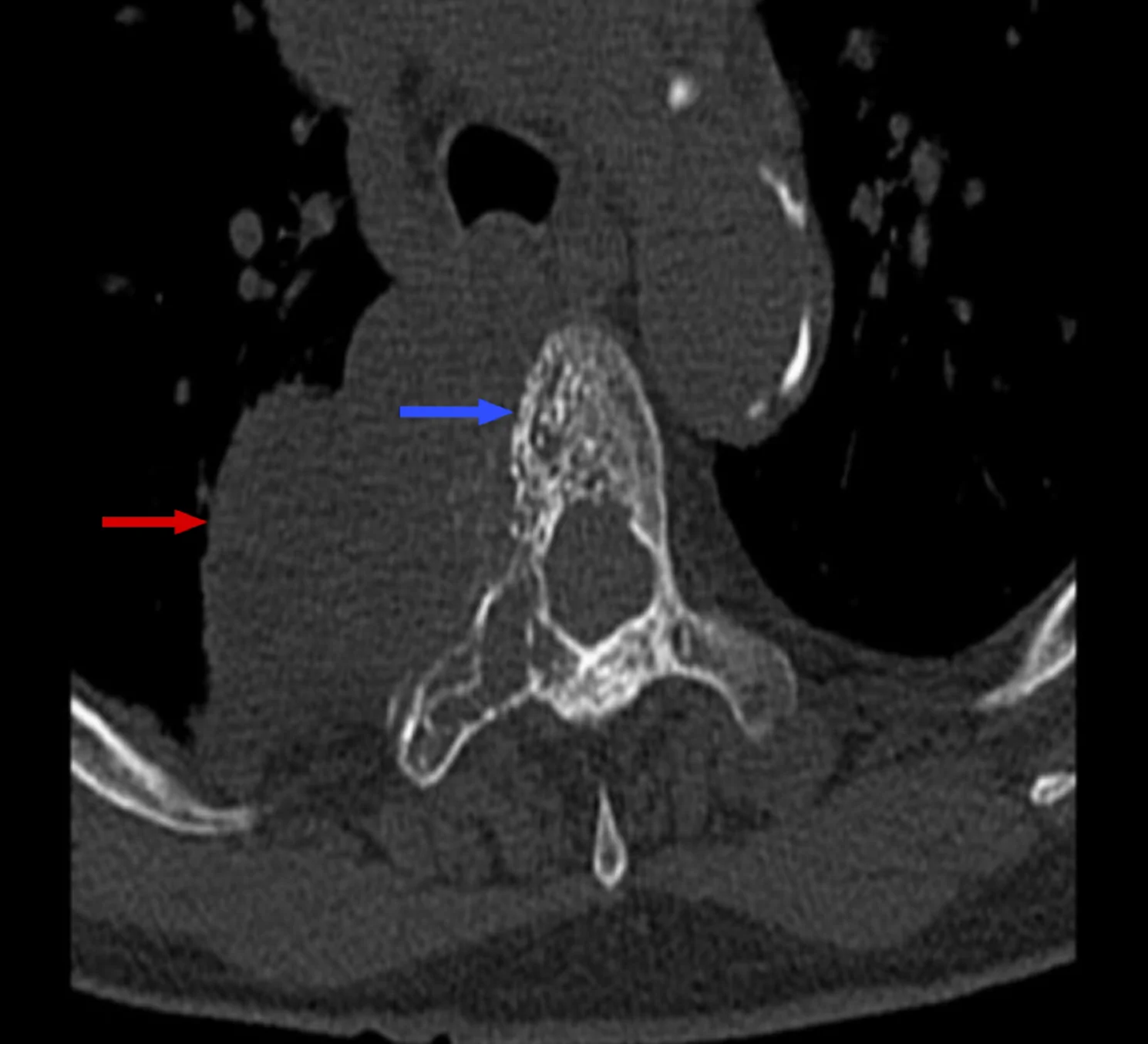
Computed tomography of the thoracic spine demonstrates a heterogeneously enhancing soft tissue lesion (Red arrow) extending from D3 to D6 with right paraspinal, extradural, and neural foraminal extension. The D4 vertebral body shows coarse, heterogeneous trabecular density and bony expansion (Blue arrow)

Magnetic resonance imaging of the thoracic spine demonstrated a well-defined, homogeneously enhancing dumbbell-shaped lesion centered at the D4 vertebral level with extension through the right neural foramen into the intrathoracic paravertebral space [Figs. 1 and 2]. The intraspinal component caused severe spinal cord compression. Computed tomography showed a characteristic “honeycomb” appearance involving the D4 vertebral body, pedicle, and lamina, suggestive of a hypervascular lesion [Figure3].

**Fig. 4:**
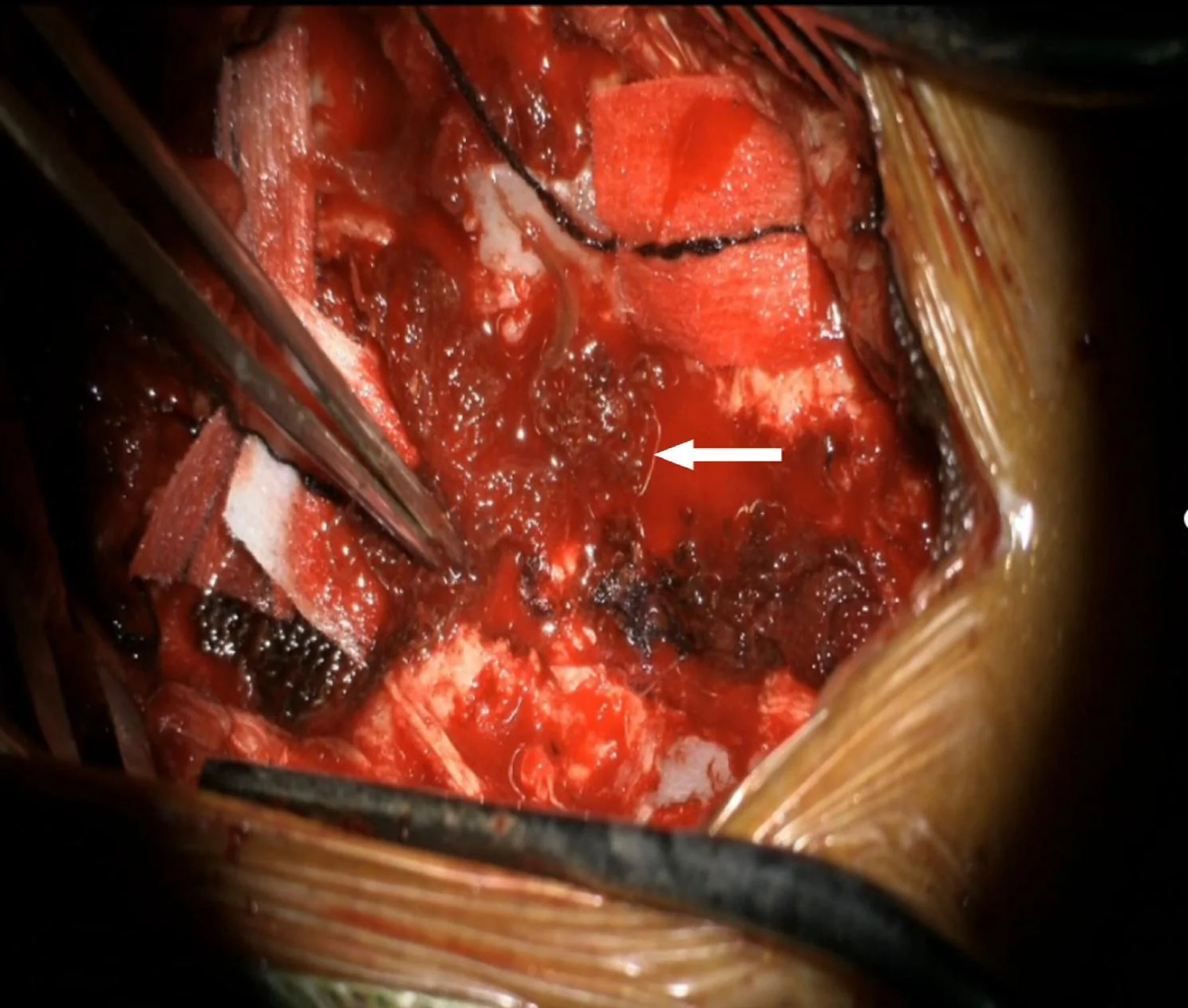
An extradural, markedly hypervascular tumor (Arrow) was encountered, appearing congested and friable with a tendency for brisk bleeding on manipulation

**Fig. 5:**
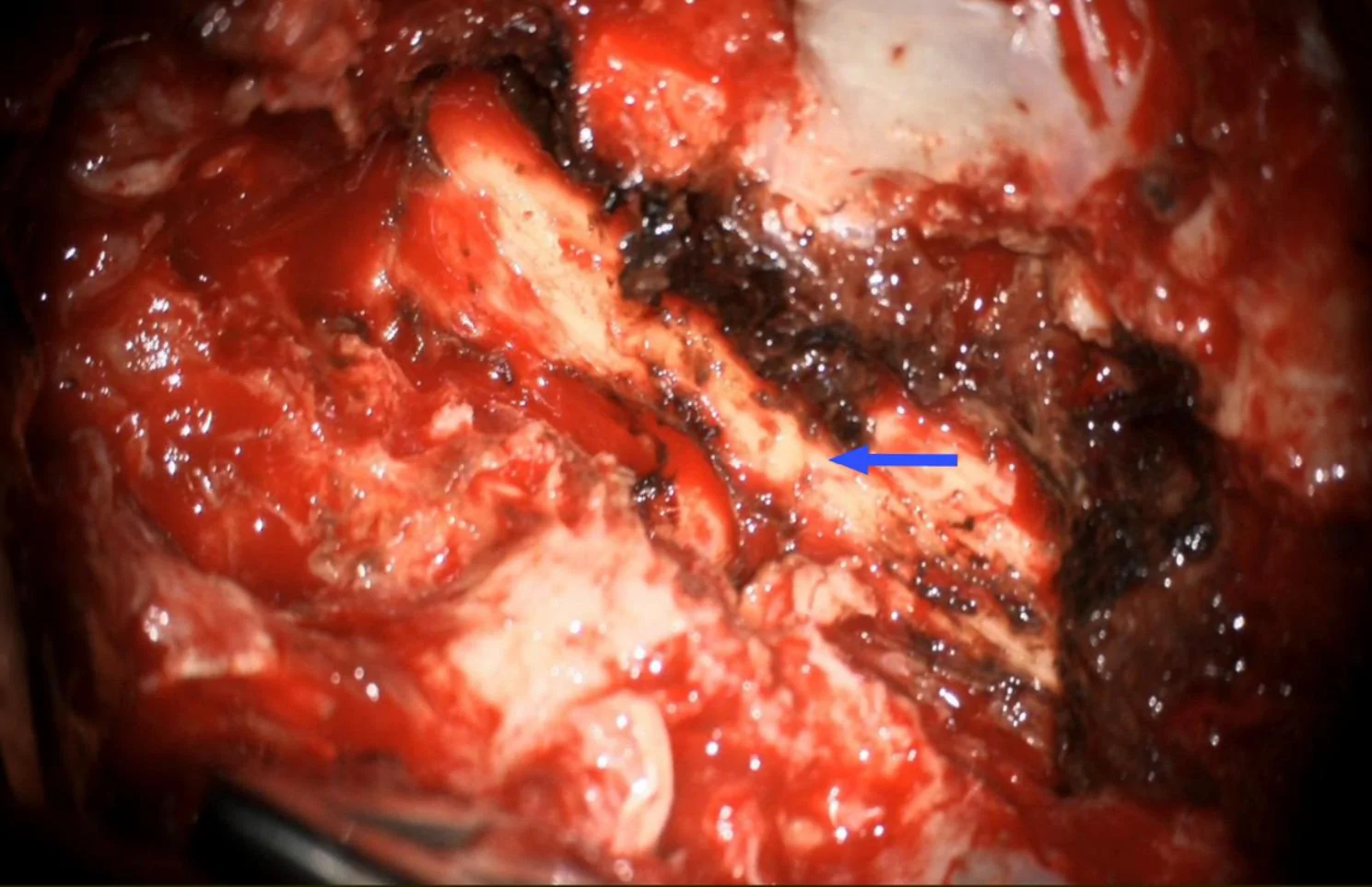
The lesion demonstrated transforaminal extension into the thoracic cavity. After complete excision of the spinal component, the underlying dura was clearly identified (Arrow) and remained intact without evidence of invasion or breach

The patient underwent D3–D5 laminectomy with planned costotransversectomy. Intraoperatively, the lesion was identified as a purely extradural, markedly hypervascular tumor with foraminal and paravertebral extension [Figure 4]. Following circumferential devascularization, the intraspinal component was excised [Figure 5]. During tumor exposure, brisk hemorrhage was encountered from the eroded D4 lamina and hypervascular epidural tumor. After excision of the intraspinal component, significant paravertebral extension remained. Owing to persistent bleeding and difficulty obtaining vascular control through the posterior approach alone, the operative strategy was modified to a right posterolateral mini-thoracotomy. The thoracic surgery team assisted the neurosurgical team in exposure and resection of the intrathoracic component. This combined approach enabled vascular control, complete tumor excision, and hemostasis.

**Fig. 6:**
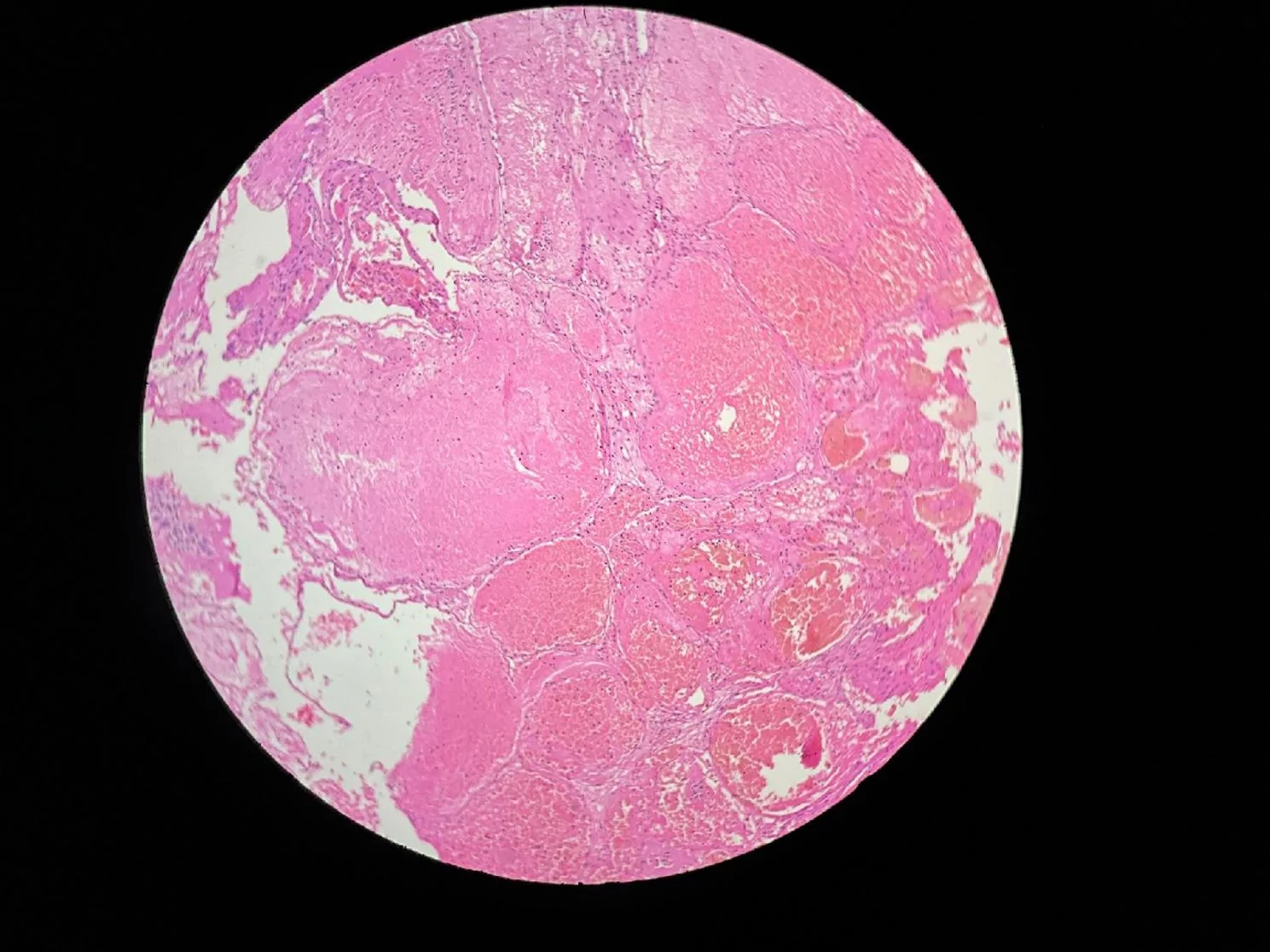
Histopathological examination. Hematoxylin and eosin staining demonstrates lobules of closely packed capillary-sized vascular channels lined by flattened endothelial cells without atypia. Thick-walled vessels with smooth muscle media are present. No mitotic figures or necrosis are identified. Findings are consistent with benign capillary hemangioma

Postoperatively, the patient showed rapid neurological improvement and was able to stand with support by postoperative day two. Histopathology demonstrated lobules of proliferating capillary-sized vessels lined by flattened endothelial cells without atypia, consistent with benign capillary hemangioma [Figure 6].

## Discussion

Dumbbell-shaped spinal tumors present a diagnostic challenge because of their diverse etiologies. Although schwannomas are the most common lesions, meningiomas, neurofibromas, ganglioneuromas, hemangioblastomas, lymphomas, Ewing’s sarcoma, and vascular tumors should also be considered [7, 8]. In the present case, homogeneous enhancement and foraminal extension initially favored a nerve sheath tumor, whereas extensive vertebral involvement with a honeycomb trabecular pattern suggested a vascular lesion. The differential diagnoses and distinguishing features are summarized in Table 1. Histopathological examination ultimately confirmed capillary hemangioma.

**Table 1:** Differential Diagnosis of Dumbbell-Shaped Thoracic Spinal Lesions

Diagnosis	Typical imaging features	Reason excluded in present case
Schwannoma	Homogeneous enhancement, neural foraminal widening, dumbbell morphology	Histopathology showed capillary hemangioma
Neurofibroma	Fusiform nerve-related mass, target sign on MRI	No nerve origin identified; pathology inconsistent
Meningioma	Dural attachment, dural tail sign	Purely extradural lesion without dural attachment
Hemangioblastoma	Highly vascular lesion, often intradural	Histology inconsistent
Malignant Peripheral Nerve Sheath Tumor	Bone destruction, heterogeneous enhancement, atypia	No atypia, mitosis, or malignant histology
Lymphoma	Homogeneous epidural soft-tissue mass	Histopathology negative
Ewing Sarcoma/PNET	Aggressive osseous destruction and soft-tissue mass	Histology inconsistent
Capillary Hemangioma	Homogeneous enhancement, hypervascularity, rare dumbbell morphology	Confirmed histologically

Most spinal epidural hemangiomas are of the cavernous type, whereas epidural capillary hemangiomas are exceedingly rare, with fewer than ten cases reported to date [7, 10]. Histologically, capillary hemangiomas are characterized by lobules of thin, endothelium-lined capillary vessels separated by fibrous septa, in contrast to cavernous hemangiomas, which consist of dilated vascular spaces [11, 12]. Among the limited reported cases of epidural capillary hemangioma, only approximately five have demonstrated a dumbbell configuration, highlighting the exceptional rarity of this entity. The clinical characteristics of these cases and comparison with the present case are summarized in Table 2.

**Table 2:** Reported Cases of Dumbbell-Shaped Spinal Epidural Capillary Hemangioma and Comparison With the Present Case

Author (Year)	Age/Sex	Location	Dumbbell Extension	Preoperative Diagnosis	Treatment	Outcome
Badinand et al. (2003) [8]	54/F	Thoracic epidural	Foraminal and paravertebral extension	Schwannoma	Surgical excision	Good neurological recovery
Hasan et al. (2011) [4]	69/F	Thoracic epidural	Dumbbell-shaped lesion	Nerve sheath tumor	Gross total excision	Favorable outcome
Yim et al. (2015) [9]	58/M	Lumbar epidural and paraspinal region	Epidural with paraspinal extension	Neurogenic tumor	Surgical excision	Good recovery
Niznick et al. (2020) [11]	73/M	Thoracic epidural	Foraminal extension	Schwannoma	Surgical excision	Neurological improvement
Wu et al. (2022) [5]	64/F	Lumbar epidural	Dumbbell morphology	Schwannoma	Gross total resection	Symptom resolution
Present case	66/F	D3–D5 thoracic epidural	Foraminal and large intrathoracic paravertebral extension	Schwannoma	D3–D5 laminectomy with right mini-thoracotomy and gross total excision	Recovery of ambulation and bladder function

MRI is the imaging modality of choice for evaluating spinal epidural lesions [6]. Capillary hemangiomas typically appear isointense on T1-weighted sequences and hyperintense on T2-weighted images, with marked homogeneous gadolinium enhancement [13, 14]. These features closely resemble those of schwannomas and meningiomas, particularly when a dumbbell morphology is present [13]. In contrast, cavernous hemangiomas often demonstrate heterogeneous signal intensity due to hemorrhage, thrombosis, or calcification [5]. In the present case, the combination of homogeneous enhancement and classic dumbbell configuration strongly favored a preoperative diagnosis of schwannoma, delaying recognition of the vascular nature of the lesion.

Definitive diagnosis relies on histopathological evaluation. Capillary hemangiomas demonstrate compact lobules of capillary-sized vessels, whereas cavernous hemangiomas show large, dilated vascular channels [11]. Immunohistochemical positivity for endothelial markers such as CD31, CD34, and Factor VIII further confirms the diagnosis [11, 14]. Despite their benign histology, capillary hemangiomas can exhibit pronounced intraoperative vascularity, resulting in substantial blood loss and operative difficulty.

The dumbbell variant of spinal capillary hemangioma has been reported across cervical, thoracic, and lumbar regions without a clear regional predilection [8, 9]. Reported patients are typically middle-aged or elderly, and surgery is frequently complicated by significant intraoperative hemorrhage. Surgical excision, with or without preoperative embolization, remains the treatment of choice [6]. When suspected preoperatively, embolization may reduce blood loss and improve surgical safety; however, this option is seldom considered because these lesions are often misdiagnosed as schwannomas.

Although capillary hemangiomas are benign, gross total excision provides the best long-term outcome and is associated with a low recurrence rate [15, 16]. Complete resection may be challenging in dumbbell lesions because of extensive foraminal or paravertebral extension [4]. When subtotal resection is unavoidable, radiosurgery has been advocated for residual or recurrent disease, although long-term outcome data remain limited [14, 17] .

In the present case, extensive osseous involvement, marked hypervascularity, and intraoperative hemodynamic instability necessitated modification of the surgical strategy, shifting from a posterior-only approach to a combined laminectomy and thoracotomy. This experience underscores the importance of maintaining a broad differential diagnosis and surgical flexibility when managing dumbbell-shaped spinal lesions.

## Conclusion

Dumbbell-shaped epidural capillary hemangioma is an exceptionally rare spinal lesion that can closely mimic schwannoma on imaging, leading to preoperative diagnostic uncertainty. Despite its benign histology, the tumor may exhibit marked hypervascularity and extensive osseous and paravertebral involvement, resulting in significant intraoperative hemorrhagic risk. Gross total excision remains the treatment of choice and is associated with favorable neurological recovery. Preoperative recognition of a possible vascular etiology, consideration of embolization, and readiness to modify the surgical approach including combined posterior and thoracic exposure are essential to ensure safe resection and optimal outcomes.

## Data Availability

No datasets were generated or analysed during the current study.

## Declarations

### Clinical trial number

Clinical trial number: not applicable.
